# The Utilization of a Coupled Electro-Thermal-Mechanical Model of High-Voltage Electric Pulse on Rock Fracture

**DOI:** 10.3390/ma16041693

**Published:** 2023-02-17

**Authors:** Weikang Feng, Pingping Rao, Sanjay Nimbalkar, Qingsheng Chen, Jifei Cui, Peihao Ouyang

**Affiliations:** 1 Department of Civil Engineering, University of Shanghai for Science and Technology, 516 Jungong Road, Shanghai 200092, China; 2 School of Civil and Environmental Engineering, University of Technology Sydney, 15 Broadway, Ultimo, Sydney, NSW 2007, Australia; 3 Technology Research Center of Ecological Road Engineering, Hubei University of Technology, Wuhan 430068, China

**Keywords:** electro-thermal-mechanical coupling, plasma channel, conductor mineral particles, rock fracture

## Abstract

Our research proposes a unique coupled electro-thermal-mechanical model that takes electric breakdown and heterogeneity into account to show the mechanism of rock fracturing under high-voltage electropulses. Using finite element numerical software, the process of high voltage electrical pulse injection into the rock interior for breakdown is described, and the formation law of plasma channels during the electrical breakdown process is comprehensively analyzed in conjunction with the conductor particles present within the rock. On the basis of electrical, thermal, and mechanical theories, a coupled multi-physical field numerical model of rock failure under the action of high-voltage electrical pulses is developed, and a random distribution model is utilized to simulate the potential occurrence of conductor particles in the rock. Innovative numerical model indicates plasma channel creation in the rock-crushing process. Prior to the formation of the plasma channel, the temperature and stress are approximately 10^3^ k and 10^−2^ MPa, respectively. Once the plasma channel is formed, the temperature and stress increase abruptly in a short time, with the temperature reaching 10^4^ k and the stress reaching 10^3^ MPa or higher. In addition, it is revealed that the breakdown field strength is the essential factor in plasma channel creation. The heterogeneity of the particles within the rock and the fluctuation in electrode settings are also significant variables influencing the creation of channels. The presented model contributes to a better understanding of the mechanism of rock fragmentation during high-voltage electrical pulses, which has substantial implications for oil exploration and mineral extraction.

## 1. Introduction

High-voltage electric pulse technology is a new drilling technology that has been developed gradually. It differs from other rock fracturing technologies such as the drill-and-blast method and the full-scale hard rock boring method in that it uses high-voltage electric pulse discharge to cause electrical breakdown of rocks or liquid media, thereby generating a shock wave with sufficient energy to damage the rocks [1]. The high-voltage electrical pulse rock-fracturing technique is now utilized in multi-disciplinary engineering domains such as rock mining [2], micro-mineral decomposition [3], and scale removal and mineral recovery [4]. In comparison with several other traditional rock-fracturing techniques, this method has advantages such as directional crushing and simple control of the crushing process, and it is known as a green rock-fracturing technology due to its rapid fracturing speed and high efficiency particularly in complex and hard rocks [5,6].

As the 1950s were ending, some Russian scholars (e.g., Vorobiev [7] and co-workers) examined the electric field strength of water and rocks, and determined the relationship between the rising edge of the pulse and the breakdown field strength of water and rocks. Under the influence of short pulses with a rising edge shorter than 500 ns, it was discovered that the rock’s breakdown field strength was less than that of the water, resulting in the electric fragmentation of the rock. When the rising edge of the pulse is greater than 500 ns, the rock’s breakdown field strength is less than that of the water, and the electrical pulse first generates a shock wave in the water, which is then transferred to the rock through the pulse energy, resulting in the liquid-electric fragmentation of the rock [8,9]. Andres [10] investigated the effects of the voltage level and energy density on granite fractures and concluded that the greater the strength of the electric field applied to the rock, the lower the energy density required to fracture the rock; three ways to optimize energy consumption were experimentally discovered: increasing the efficiency of the pulse generator, optimizing the geometry of the electrodes, and optimizing the waveform of the electrical pulse [9]. For this purpose, Inoue et al. [11], Bluhm et al. [12], Biela et al. [13], Vizir et al. [14], and He et al. [15] created a high-voltage pulse power supply and an upgraded magnetic switch, respectively. Wielen et al. [16] conducted pulse discharge studies on 20 rocks using a SelFrag high-voltage pulse crusher, and the results of the study demonstrated that the number of discharges and the voltage are the two most influential parameters in determining the ultimate product size. In addition to field experiments on rocks, numerical simulation analysis is increasingly being incorporated into related research. Li et al. [17] analyzed the impacts of granite composition and electrical parameters on high-voltage electrical pulses and deduced the distribution of the electric field within the rocks under the influence of electrical pulses. Most models are developed for insulators; therefore, they are unsuitable for non-homogeneous media such as rocks. Neglecting the influence of electrical structure parameters on the breakdown path of rock media, Zhu et al. [18] developed a coupling between the electric breakdown process and the circuit structure parameters by controlling the circuit current and the loading voltage in the current field controlled by the circuit. Cho et al. [19] studied the high-voltage electric pulse under the rock fracture process and concluded that the fracture effect of high-voltage pulse discharge on rocks is caused by dielectric breakdown-induced body forces. The results demonstrated that the rock fracture process is affected by rock inhomogeneity and external conditions (e.g., loading rate). In accordance with the classical explosion theory, rocks are modeled as homogeneous, isotropic, and incompressible fluids. Burkin et al. [20,21] and Kuznetsova et al. [22,23] constructed some dynamic models of electrobursting of rocks under the influence of electrical impulses and obtained numerical simulations of the fluctuation of parameters including current, voltage, and shock wave pressure. The physical process of electro pulse boring (EPB), however, cannot be well simulated or explained. Using finite elements, Rao et al. [24] modeled the damage process and mechanism of rocks under the influence of a high-voltage electric pulse. Through the measured data and 3D numerical simulation, Cui et al. [25] analyzed the deformation of the retaining structure and surrounding strata.

Zhang et al. [26] determined the link between breakdown probability and breakdown field strength for yellow sandstone during the process of high voltage electro pulse (HVEP) rock fracturing. Based on the test, when the internal field strength of the rock is less than the starting breakdown field strength, the probability of breakdown is very low because the energy inside the rock is too small to form a plasma channel, resulting in a very low probability of breakdown regardless of whether the rock breakdown occurs or not. The occurrence of rock fracture is connected to the creation of plasma channels. Rakotonandrasana et al. [27] established a model for flowline development based on the fundamental process of plasma production through model validation and experimental data. In addition, some models [28,29] have explored the effect of space charge and dielectric property heterogeneities on the breakdown route. Burki et al. [20] performed simulations of electrical pulses on solid materials and discovered that the depth of the plasma channel generated in rocks is typically one-third of the electrode spacing. L. Niemeyer and Wiesmann proposed a dielectric breakdown model (DBM) that introduced the concepts of threshold field strength, Ec, and falling field strength, Ed, inside the channel, while describing EBP in terms of both electric field strength factor and electric breakdown stochastic factor using an electric tree fractal theory model [30,31]. However, they did not consider the case of changing conductivity and continuous increase in energy release. On the basis of uniform channel radius [32,33], Kirchhoff’s equation, energy conservation, and other theories [23], Li et al. [34,35] calculated the mechanics of electric pulse rock-fracturing impact following plasma channel development. Extremely complex and frequently accompanied by electric-thermal-force multi-field coupling, the EPB procedure is characterized by a high degree of complication. However, in the aforementioned models, neither the plasma channel generation process nor the heterogeneity of the rock nor the alteration of electrical characteristics throughout the breaking process are taken into account. There is no model accurate enough to mimic the production of plasma channels, which is typically achieved by experimental instrumentation scanning, but cannot explain the process; furthermore, there is no study that explains the principle of ion channel formation during rock disintegration. Even if the electrode is in direct contact with the rock during the electric pulse breaking process, the interior composition of the rock will also undergo a significant change in composition conductivity and exert some impact on the development of plasma channels during the breaking process.

There is a need for precise models exist to describe the creation of plasma channels during electrical pulses. Some previous models do not account for the potential presence of conductor particles in the rock, and do not provide a visual representation of the formation of plasma channels. Due to the interaction of many physical fields during the breaking process and the variability of the rock material, it is especially challenging to simulate rock fracture. In addition, the process of rock fracturing is frequently completed within a few hundred nanoseconds and the creation of interior plasma channels cannot be well described. In order to characterize the mechanism of rock fracturing under the influence of high-voltage electric pulses, a breakdown model was developed that implements the coupling of electro-thermal-mechanical multi-physical fields and takes particle heterogeneity into consideration. The model explains the relevant factors influencing the formation of plasma channels and how to better form plasma channels within the rock. Moreover, our research provides an effective method for the optimization of parameters in the electric pulse process and the development of rock-fracturing tools, which has significant engineering implications for enhancing the rock-fracturing efficiency.

## 2. Analysis of the Mechanism of Rock Fracturing by High-Voltage Electric Pulse

The impact process of an electric pulse on rocks is a multi-physical field coupling process that primarily consists of an electro-thermal-mechanical coupling effect. When the electric pulse reaches the interior of the rock in the form of electric current, the rock is rapidly heated by the Joule effect. The electric pulse discharge will generate an arc channel inside the rock, thereby generating a plasma with rapid fluctuations in temperature and density in the channel. This plasma will cause the arc channel to rapidly expand and squeeze the surrounding medium, thereby generating shock wave pressure. The associated release of huge amounts of heat in the arc channel modifies the shift in phase transition at the channel, hence influencing the electric shock damage response of the entire structure. The effect of the pulse on the rock is dependent on both the course of the pulse through the rock and the subsequent mechanical behavior resulting from the substantial thermal expansion generated by the high-voltage pulse acting on the rock. Although these processes are interrelated, they operate on distinct time scales and are modeled individually.

### 2.1. Conceptual Model of High Voltage Electrical Pulse Rock Fracturing

**Figure 1:**
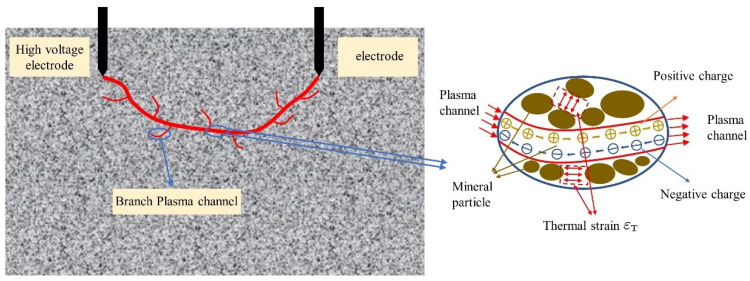
Conceptual model of the rock fracturing under high-voltage electropulse.

Figure 1 provides a conceptual model of rock fracture under high-voltage electropulse. The high-voltage electrode end supplies the high-voltage electrical pulse. When the electrode strength is sufficient, the internal field strength of the rock surpasses its breakdown field strength and a plasma channel between the high-voltage electrode and the ground electrode is created within the rock. As depicted in the figure, when the electrode is connected, a current density aggregation will occur at the electrode’s extremity due to the enormous potential difference, causing positive and negative charges within the rock to shift. As the electrode voltage continues to be applied, the plasma channel is produced gradually. As depicted in the illustration below, the final channel trajectory of the plasma channel is the trajectory of the charge movement. If the applied electrode voltage or current is excessive, additional branching channels are also derived around this channel, some branch directly from the electrode and others branching out through the primary channel to neighboring channels, as outlined in the conceptual model. When the channel is fully created, subsequent energy transmission occurs directly. During this process, the temperature at the channel rises significantly, and thermal expansion stresses resulting from the high temperature operate on the walls of the channel and on the interaction between rock particles. As the energy continues to increase, rock fracture occurs when the stress intensity surpasses the rock’s critical tensile strength; this is the full conceptual model of high-voltage electrical pulse rock fracturing.

### 2.2. Electric Field Model

Under transient action, the current field is controlled within the rock by Maxwell’s first equation:

(1) $$\nabla \cdot J=\frac{\partial \rho_{d}}{\partial t}$$

(2) $$J=J_{c}+J_{d}$$

(3) E=−∇V

where *E* denotes electric field strength; D denotes potential shift; *V* denotes potential; J denotes full current density; $J_{c}$ denotes conduction current; $J_{d}$ denotes displacement current, and $J_{d}=\frac{\partial D}{\partial t}$; and $\rho_{d}$ is charge density. From the intrinsic relationship of the electromagnetic field, we know that:

(4) $$J_{c}=\sigma_{c}E$$

(5) $$D=\varepsilon_{0}\varepsilon_{r}E$$

where, *D* denotes potential shift; $\sigma_{c}$ is the conductivity; $\varepsilon_{0}$ is the vacuum permittivity with a value of 8.854 × 10^−12^ F/m; $\varepsilon_{r}$ is the relative permittivity of the medium (rock, electrode, among others). The vector *J* full current density, due to its dispersion is constant to 0, ∇J=0, and then combined with the above equations, the controlling equation of the current field can be transformed into an equation controlled by the potential:

(6) $$\sigma_{c}\nabla^{2}V-\varepsilon_{0}\varepsilon_{r}\nabla^{2}\frac{\partial V}{\partial t}=0$$

**Figure 2:**
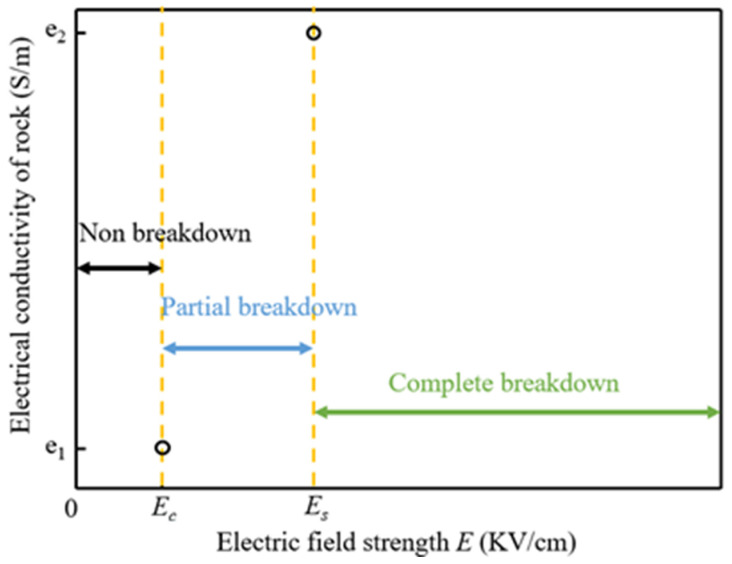
Variation of electrical conductivity of rock with electric field strength.

It is evident from Equation (6) that the source of electric heat is primarily related to the potential and conductivity; while the potential may be inferred from the effect of the electric current, the major concern is how to calculate the conductivity’s magnitude. The initial conductivity of the rock is estimated to be 4 × 10^−5^ S/m based on the material’s own characteristics. After a substantial amount of heat is delivered into the rock, the rock’s condition begins to alter near the electrode. The magnitude of conductivity is the most essential element impacting the conduction of the current, so we disregard the changes of other parameters in this process and focus on the state change of conductivity. According to the research of Zhu et al. [18], when the field strength of the internal portion of the rock reaches the breakdown field strength, the electrical properties of this portion of the rock begin to change from resistance to conductor, and the conductivity at the channel can reach 1e6 at this point. Based on this occurrence, we graphically depict the conductivity change process as shown in Figure 2. *Ec* and *Es* refer to the starting breakdown field strength and complete breakdown field strength of the rock in S/m. When the internal field strength of the rock reaches *Ec*, the rock starts to undergo a state change and conductivity begins to increase; when *Es* is reached, it means that the channel has been formed and is completely broken through.

### 2.3. Temperature Field Model

According to Maxwell’s first equation, the current consists of a conduction current and displacement current. The nature of displacement current, according to the theory of electromagnetism, is a changing electric field whereas the nature of conduction current is the directional movement of free charge; therefore, the conduction current produces Joule heat when passing through a conductor, whereas the displacement current does not produce Joule heat. When the pulse current acts on the electrode, the current acts on the rock as a conduction current and produces a significant amount of heat into the interior of the rock. The source of heat is mostly the conduction current, as described by the following equation:

(7) $$Q=J_{c}E$$

Then bringing Equations (3) and (4) into (7), we get that:

(8) $$Q={\left(-\nabla V\right)}^{2}\sigma_{c}={\left(\nabla V\right)}^{2}\sigma_{c}$$

According to the law of conservation of energy, the expression of the rock heat conduction equation in the process of rock fracturing can be established:

(9) $$\rho c_{\rho }\frac{\partial T}{\partial t}-\nabla (\lambda \Delta T)=Q$$

where: $\rho c_{\rho }$ is the heat capacity of the soil; λ is the heat conduction coefficient; Q is the heat source, which in this paper is mainly the heat generated by the resistance of the rock body. Combining Equations (9) and (10) can be reduced:

(10) $$\rho c_{\rho }\frac{\partial T}{\partial t}-\nabla (\lambda \Delta T)={\left(\nabla V\right)}^{2}\sigma_{c}$$

The temperature changes drastically during rock fracturing. A transition from phase 1 to phase 2 occurs. When phase change is considered in solid materials, the density is defined on the material frame. Therefore, a single density should be defined for the different phases to ensure mass conservation on the material frame:

(11) $$\rho =\rho_{1}=\rho_{2}$$

The expression of the specific enthalpy, *H*, can be simplified:

(12) $$H=\theta H_{1}+(1-\theta )H_{2}$$

The apparent heat capacity, *C_p_*, used in the heat equation, is given by:

(13) $$C_{P}=\theta_{1}C_{p,1}+\theta_{2}C_{p,2}+L_{1\to 2}\frac{\partial \alpha_{m}}{\partial T}$$

where $L_{1\to 2}$ is the Latent heat from phase 1 to phase 2; the mass fraction is:

(14) $$\left\{\begin{array}{l} \alpha_{m}=\frac{1}{2}\frac{\theta_{2}-\theta_{1}}{\theta_{1}+\theta_{2}}\\ \theta_{1}+\theta_{2}=1 \end{array}\right.$$

### 2.4. Mechanics Field Model

During the gradual formation of the plasma channel, the temperature inside the channel can reach more than 10^4^ k, and the impact force due to thermal expansion can reach 1 Gpa, which is much larger than the uniaxial tensile strength of the rock (~10 MPa), so the shock wave caused by thermal expansion is sufficient to cause damage inside the rock and cause rupture. In the process of fracturing rock by electric pulse, when considering the heat transfer effect of solid breakdown, due to the short time of breakdown, taking the elastic-brittle rock as an example, the stress-strain relationship can be considered in form of linear elasticity, while the material satisfies the following relationships within small strain domain:

(15) $$\left\{\begin{array}{l} \begin{array}{l} \varepsilon =\frac{1}{2}\left[{\left(\nabla u\right)}^{T}+\nabla u\right]\\ C=C(E,\nu ) \end{array}\\ \sigma =C:\varepsilon \end{array}\right.$$

where *u* is the displacement field variable; σ is the thermal stress (Pa); *C* is the isotropic elastic matrix, controlled by Young’s modulus and Poisson’s ratio; *ε* is the strain, denoted as:

(16) $$\varepsilon =\varepsilon_{e}+\varepsilon_{T}$$

where ε, $\varepsilon_{e}$, and $\varepsilon_{T}$ are the tensor of total strain, effective strain, and thermal strain. The temperature rise may cause the thermal strain,$\varepsilon_{T}$ expressed as:

(17) $$\varepsilon_{T}=\alpha (T)(T(t)-T_{0})$$

where, α(T) is the coefficient of thermal expansion, (k − 1); *T*_0_ is the initial temperature (k); *T(t)* is the plasma channel temperature (k).

### 2.5. Relationship between the Physical Fields

**Figure 3:**
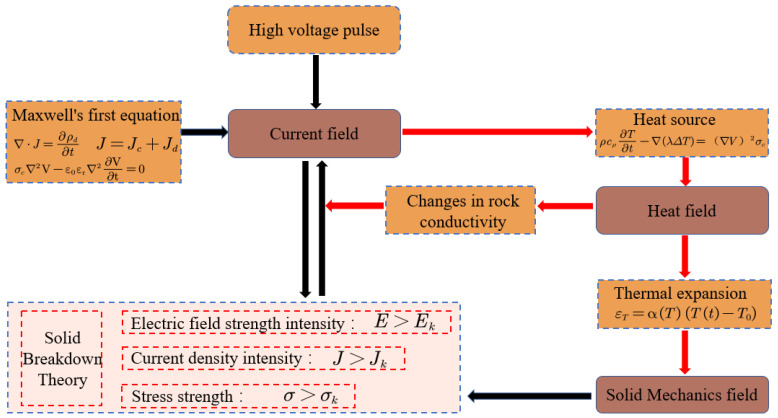
Electro-thermal-mechanical multi-physical field coupling relationship.

The process of EPB implementation involves electric, temperature, and mechanical fields. Therefore, the process of EPB implementation is complex with multiphase and Multiphysical field changes. The relationship between the electro-thermal-mechanical coupling in the above-mentioned electric pulse process can be roughly represented by Figure 3.

## 3. Model Simulations

### 3.1. Construction of Geometry

**Figure 4:**
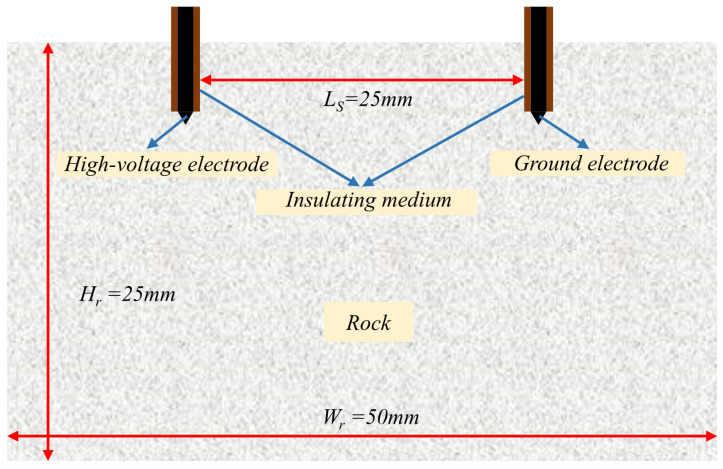
Geometric pattern of rock in electric pulse fracturing.

In our paper, we refer to Timoshkin et al. [36], Kusaiynov et al. [37], Yudin et al. [38], and Li et al. to design the structure of electrode drill bits [39,40], which consist of one or more pairs of electrodes and are generally placed in parallel, and we use the rock-fracturing unit of EPB drill bits to develop a two-dimensional high-voltage electrical pulse penetration model. In this research, the same electrode pair method is utilized, as depicted in Figure 4. The software used in our study is a commercial software, which is a comprehensive software of multi-physics field coupling. In the model, the high-voltage electrode and the ground electrode are parallel and positioned on the same side of the rock. To avoid the interference of reflected waves and imitate the actual conditions of the electric pulse rock experiment, the model border is considered to have a low reflection coefficient. The assumption is that the material of both electrodes is a perfect conductor and that the electrodes are encased in an insulating liquid. Note that the breakdown strength of the insulating jacket must exceed that of the hard rock in order to prevent the breakdown from occurring outside the rock. Table 1 displays the design of the model-related parameters. In the simulations investigated, both the type of hard rock and the distance between electrodes can be used as variable parameters to determine the effect of the relevant parameters on hard rock fragmentation. Table 2 [18] lists the material parameters of the electrodes and the insulating media surrounding the electrodes.

### 3.2. Selection of Basic Parameters of Rock

Table 3 shows the basic parameters of the rock selected for our paper [18]. The effect of parameters such as conductivity and relative permittivity on the formation of plasma channels is investigated.

**Figure 5:**
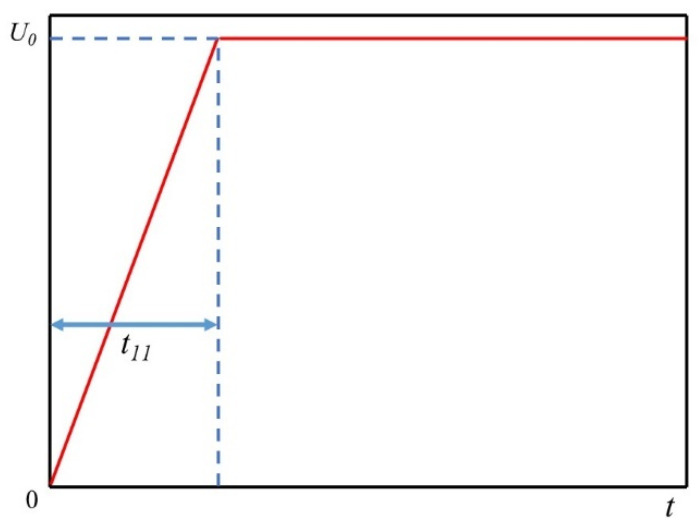
The voltage pulse process.

In the simulations in our paper, the high voltage electrode extreme input is a high voltage electrical pulse, and the pulse voltage variation is shown in Figure 5. *U*_0_ refers to the peak value of the pulse voltage and *t*_11_ refers to the pulse time for the electrode voltage to reach the peak value. Depending on the peak and pulse time, there is also a certain influence on the formation of plasma channels in the rock fracturing process which, in turn, affects the issue of the efficiency of the electric pulse fracturing, and the specific influence law is shown in the Results and Discussion section.

### 3.3. Distribution of Conductor Particles

Yuansheng Li [41] found, through numerical simulations, that pyrite plays an effect of directing current breakdown inside the rock. For this reason, to gain more insight into the effect played by mineral particles inside the rock on the electric pulse breaking, we assume a two-dimensional rectangular rock domain with dimensions as described in Section 3.1. We embedded different mineral particles in the rock to consider the effect of the heterogeneity of the rock particles on the formation of plasma channels during the electric pulse breaking process. Two types of particles are set up in our paper belonging to two systems of particle groups, constituted as ellipses and circles inside the rock, systematically homogeneous and with no mutual contact between the particles. The particles are assumed to have constant dimensional size, the ellipse being 1.5 mm on the long axis and 1 mm on the short axis; the circle has a radius of 1 mm. The distribution of particles in the rock is referred to Carl E. Renshaw’s definition of fracture in rock, and the density distribution of particles satisfies the following [42]:

(18) $$\rho =\frac{1}{A^{2}}\sum_{i=1}^{n}\frac{\left(\pi a_{i}b_{i}\right)}{2}+\frac{1}{A^{2}}\sum_{j=1}^{m}\frac{\pi a_{j}{}^{2}}{2}$$

where *n* and *m* are the number of elliptical and circular particles, respectively, which are variable and determine the particle density; *A* is the area of the rock domain; *a_i_* and *b_i_* are the long and short semi-axis lengths of the ellipse, respectively; *a_j_* is the radius of the circle. *ρ* is the density distribution of the particles, which is taken as 0.17 in our paper, and the distribution is shown in Section 3.4.

### 3.4. Setting of Grid and Tolerance

**Figure 6:**
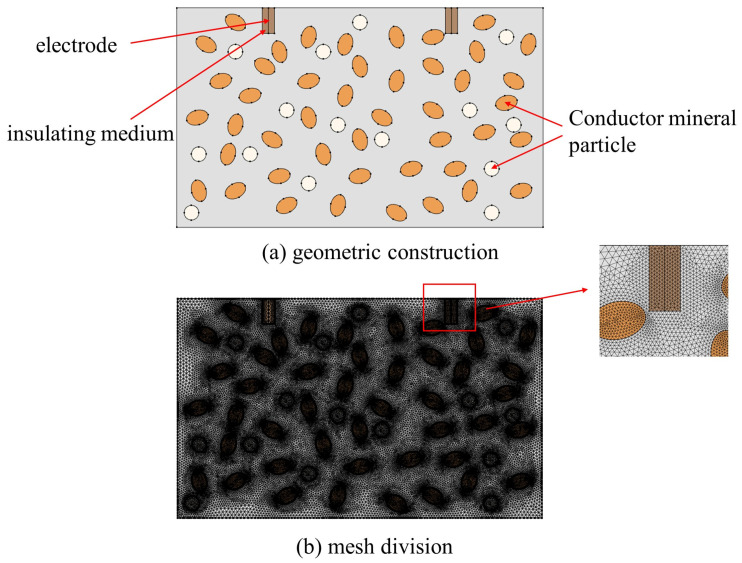
Geometric construction and mesh division of the model: (**a**) geometric construction, (**b**) mesh division.

In order to enhance the convergence of the model calculation and the accuracy of the model’s output, as shown in Figure 6, each domain of the model is spatially discretized using an unstructured triangular grid with a minimum cell mass of 0.4143 and an average grid mass of 0.9441, which is close to 1. The grid is of high quality. In the finite elementsoftware, there are two types of solvers: display and implicit. The solver type used in our study is the implicit solver. The finite element software has two main methods of implicit solvers, the backward difference formulation (BDF) and the generalized α. BDF is usually more stable and generalized than the generalized α, and also consider the damping case. The nonlinear case is also considered in our study. Therefore, the BDF method is used for the solution. As a consequence, the time-domain calculation of the model is performed using the BDF approach and an implicit solver. The model is configured with an initial time step of t = 10^−9^ ns and a relative error tolerance of 0.01 to assure numerical convergence and stability. Specifically, at each time step, up to 100 iterations are conducted until the relative error is less than the tolerance, at which point the solver begins to advance to the next time step. If the tolerance condition cannot be met, the solver will automatically halve the time step and recalculate until convergence is achieved. If the tolerance continues to be exceeded, the time step will be lowered. There are two methods to consider nonlinearity in the fully coupled case, one is automatic Newton and the other is constant Newton. The latter can implement the damped Newton method. This is appropriate in the case of considering nonlinearity. The backwards Eulerian method is mainly an initialization method for the singular matrices that appear during the model solving process. It is also an approach taken to improve the convergence of the model. The initial step fraction is set to 0.001, and the error estimation rules out the use of algebra to improve the model’s accuracy. Since this model incorporates the complex problem of electric-thermal-force multi-physical fields, the full coupling method is used to couple each physical field to ensure that the physical fields of the entire breakdown process are interconnected and influence one another to improve the accuracy of the calculation results. The research solution approach is a transient study with a 500 ns time step.

### 3.5. Steps of Model Calculation

Equations (6), (10) and (17) form a set of control equations for the electric-thermal-force multifield coupling of a rock under a high-voltage electrical pulse, where the fundamental unknowns are the potential *φ(t)*, temperature *T(t)* and displacement *u(t)*. Assume that the spatial domain is Ω and its boundary is Γ. Then, the idea of solving the multi-physics field coupled problem in this paper is to find a set of solutions with *φ(t)*, *T(t)* and *u(t)* as the basic unknowns in the space domain and time domain [0, *τ*], so that they satisfy the set of control equations consisting of Equations (6), (10) and (17) within the time interval of [0, *τ*] as follows Initial condition:

(19) $$\left\{\begin{array}{c} \varphi (x,0)=\varphi_{0,}\forall x\in \Omega \\ T(x,0)=T_{0,}\forall x\in \Omega \\ u(x,0)=u_{0,}\forall x\in \Omega \end{array}\right.$$

The following first class boundary conditions are satisfied on Γ:

(20) $$\left\{\begin{array}{c} \varphi ={\bar{\varphi }}_{,}\forall x\in \Gamma_{\varphi }\\ T={\bar{T}}_{,}\forall x\in \Gamma_{T}\\ u={\bar{u}}_{,}\forall x\in \Gamma_{u} \end{array}\right.$$

and second class boundary conditions:

(21) $$\left\{\begin{array}{l} (\frac{\partial D}{\partial t}-\sigma_{c}\cdot \nabla V)\cdot n=0,\forall x\in \Gamma^{q}{}_{\varphi }\\ -(K\cdot \nabla T)\cdot n=\alpha (T-T_{0}),\forall x\in \Gamma^{q}{}_{T}\\ -\sigma \cdot n=\bar{t},\forall x\in \Gamma^{q}{}_{\sigma } \end{array}\right.$$

In Equations (19)–(21): *φ*(t), *T*(*t*) and *u*(*t*) are the initial voltage, initial temperature and initial displacement, respectively, and are the known voltage and current flux boundaries, respectively, and are the known temperature and heat flux boundaries, respectively,; and are the known displacement and stress boundaries, respectively.

Let the matrix of shape functions be N. The basic unknowns φ(t), T(t) and u(t) to be solved are applied in the same interpolation mode, then:

(22) $$\left\{\begin{array}{c} \varphi (x,t)=N\hat{\varphi }{\left(x,t\right)}_{,}\forall x\in \Omega \\ T(x,t)=N\hat{T}{\left(x,t\right)}_{,}\forall x\in \Omega \\ u(x,t)=N\hat{u}{\left(x,t\right)}_{,}\forall x\in \Omega \end{array}\right.$$

where: $\hat{\varphi }(x,t)$, $\hat{T}(x,t)$, $\hat{u}(x,t)$ are the voltage vector, temperature vector and displacement vector of the finite element node at time t, respectively.

The set of control partial differential Equations (6), (10) and (17) are discretized and combined with the boundary conditions (21) and (22) to obtain the set of finite element governing equations:

(23) $$C\dot{X}+KX=F$$

where: $X=\left\{\hat{\varphi },\hat{T},\hat{u}\right\}\frac{dy}{dx}$ denotes the vector of nodal values of the unknown variables, package potential, temperature, and displacement; *C*, *K*, and *F* are the coefficient matrices of the set of finite element governing equations and the vector of right end terms, respectively.

Using the finite difference method for the time domain, the iterative computation format of the time step can be obtained:

(24) $$\dot{X}=\frac{X^{i+1}-X^{i}}{\Delta t}X=\theta X^{i+1}+(1-\theta )X^{i}$$

where: *i* is the time step, *t* is the time step from time step *i* to time step *i +* 1, and θ is the integration constant. When θ = 0, the forward differential format; when θ = 1, the backward differential format; when θ = 0.5, the central differential format; and when θ = 2/3, the Galerkin differential format.

Substituting Equation (24) into Equation (23), the finite element iteration format of the above coupling process is obtained:

(25) $$(C+\theta \Delta tK)X^{i+1}=\Delta tF+[C-(1-\theta )\Delta tK]X^{i}$$

Equations (19) to (21) consider the influence of the coupling effect between each process on the boundary conditions, which can be expressed as a function of time and space, and the model is solved by the above method.

## 4. Results and Analysis

### 4.1. Full Process of Rock Fracturing under Electric Pulse

**Figure 7:**
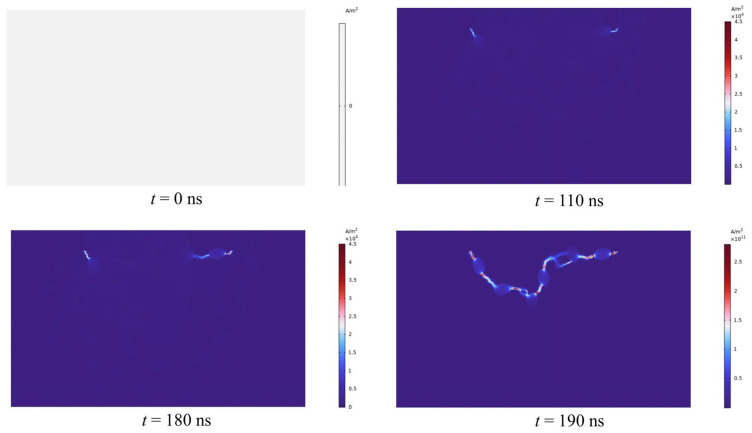
The formation of plasma channels in variable time (ns).

Figure 7 depicts the entire plasma channel creation procedure of the rock. The peak electrode voltage is 28 kv when a 200 ns pulse is delivered, and the particle density is 0.17. The high-voltage electric pulse’s rock-fracturing process may be loosely split into three stages, namely the pre-breakdown stage, the breakdown stage, and the rock-fracturing stage, as determined by simulation findings. When the pulse period is 110 ns, the rock has not yet reached its penetration field strength within the rock, and the intensity of the current density is focused around the electrode; the closer the electrode, the greater the current density. When the pulse duration reaches 110 ns, the field intensity at the electrode exceeds the breakdown field strength and the formation of the initial plasma channel commences. 180 ns later, the channel lengthens and grows further. When the rock is subjected to an electric pulse for 190 ns, the electrode voltage grows steadily, the channel forms swiftly, and a complete plasma channel is produced. The high voltage current then travels along this path of breakdown. Since the interior of the rock has been struck, the stricken location within the rock has a high concentration of current density. According to the simulation results, the values of the high-voltage pulse current in the unaffected regions are very modest or even negligible compared with the channel’s current density. Following the channel is established and the electrode voltage continues to climb; the intensity of the current density does not vary, and the magnitude is the same as at 190 ns, indicating that the current density inside the rock has achieved its maximum value after the breakdown.

**Figure 8:**
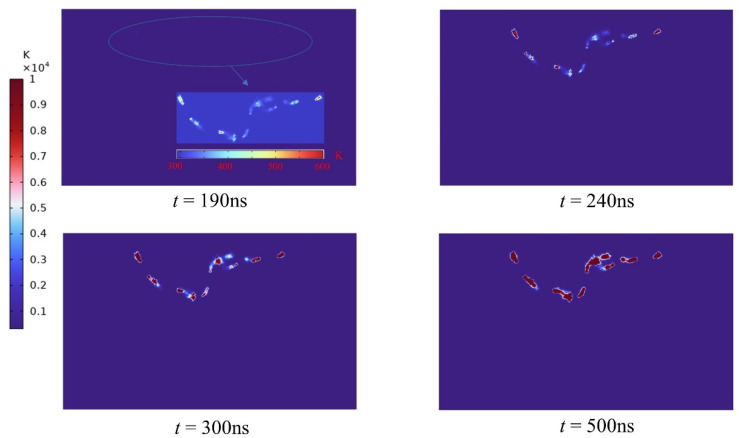
Temperature variation of the plasma channel during the electrical pulse.

**Figure 9:**
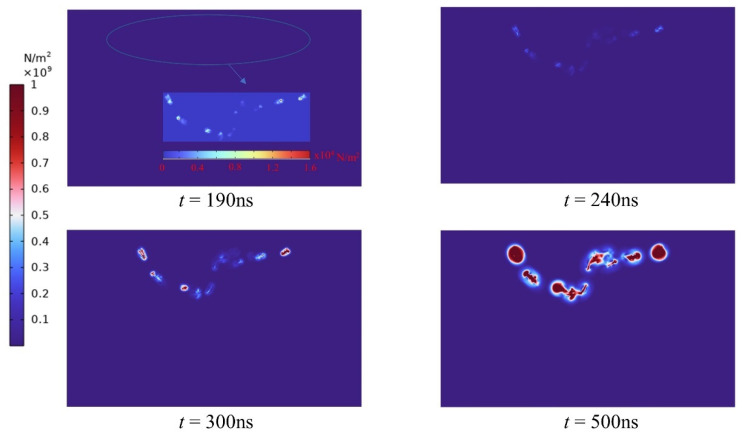
Stress changes in the plasma channel during the electrical pulse.

From the simulation results, it can be determined that the changes in temperature and stress are focused precisely at the channel, indicating that it is correct and very illustrative to characterize the plasma channel by the current density intensity. Nevertheless, when the plasma channel is formed, the resulting changes in temperature and thermal stress within the rock are inconsistent with the alterations in current density. Prior to 190 ns, when the plasma channel is not fully established, temperature and stress variations are minimal. The cloud plot of the changes in temperature and stress after the development of the plasma channel is depicted in Figure 8 and Figure 9. Unlike the intensity of the current density, the temperature rises abruptly to 10^3^ K after the formation of the channel and with the continued application of the pulse voltage, the temperature rises to 10^5^ K. When the channel is developed, the stress at the channel initially rises abruptly to approximately 10^−2^ Mpa, when the rock’s tensile strength has not yet been attained. At 200 ns, the channel stress increases to 27 Mpa. At 300 ns, the stress hits 1 Gpa, which is significantly greater than the rock’s critical stress, and the rock cracks. According to this occurrence, the moment when the rock’s temperature and tension drastically increase is when the plasma channel forms. When the channel is formed, the energies along this discharge channel will release a large amount of energy into it, causing the temperature to rise and the thermal expansion stress to increase and exert force on the surrounding rocks; when the resulting stress exceeds the rock’s own strength, the rock will be damaged and eventually fracture [43]. Consequently, the development of plasma channels is the key to high-voltage electrical pulse rock fracturing. Enhancing the creation of plasma channels can increase the effectiveness of rock fracturing.

### 4.2. The Relationship between Breakdown Field Strength and Electrical Parameters

**Figure 10:**
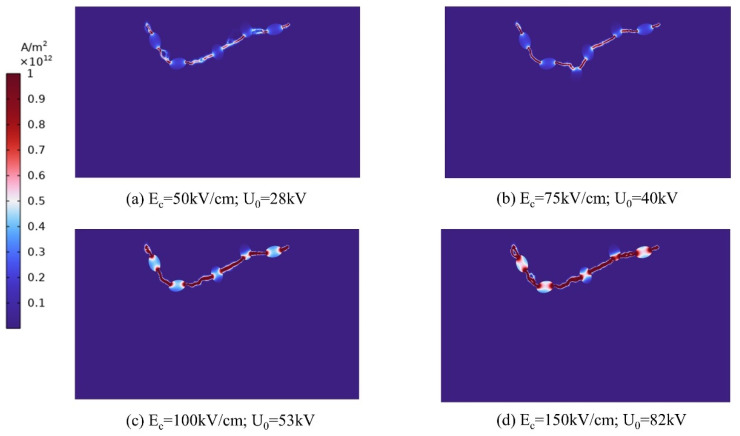
Plasma channel formation for different breakdown field strengths at corresponding electrode voltages.

According to the preceding discussion, for solid breakdown to occur in rocks the electric field strength within the rock must attain the critical electric field strength. Zhang et al. [26] determined the relationship between the breakdown law and electric field strength of yellow sandstone through experimentation. From this, it is known that there are two important breakdown strengths for different rocks, the starting breakdown field strength and the complete breakdown field strength, and that the different electrical parameters will have some effect on the rock fracturing process. Various electrical characteristics influence the fracturing procedure. Both the starting breakdown field strength and the electrical properties of the circuit module are distinct. Therefore, the size of the electrode voltage is crucial for fracturing the rock to meet the breakdown field strength of the rock in order to generate a plasma channel; however, it cannot be excessively high or the economic benefits cannot be assured. The electrode voltage rise along time is set to zero, the electrical parameters, electrode spacing, conductor particle position, and conductor particle density are left intact, and the link between breakdown field strength and electrode voltage is examined below. The breakdown strength of the rock is set to four different values of 50, 75, 100, and 150 kv/cm, respectively, to simulate the breakdown voltage of the generated plasma channels. Only one plasma channel is formed as the minimum breakdown voltage, and the current density intensity distribution under the minimum breakdown voltage corresponding to the four breakdown strengths is depicted in Figure 10.

**Figure 11:**
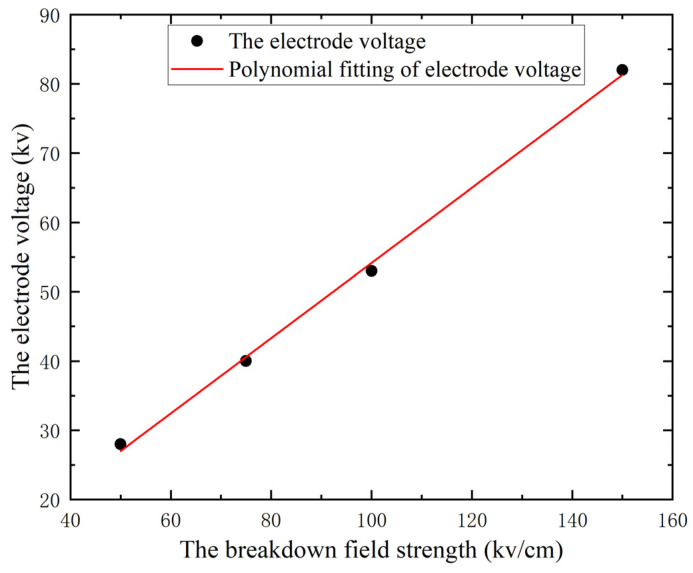
Data fit for different breakdown field strengths and electrode voltages.

As demonstrated in Figure 11, when the results of the simulated data are fitted to the data, a positive correlation between the electrode voltage and the breakdown field strength is observed. If the rock’s breakdown field strength is doubled, the applied electrode voltage must likewise be doubled in order to create a breakdown. This is because the breakdown field strength of each rock material is known, as is the minimum breakdown voltage. As long as the voltage operating on the rock does not approach its breakdown voltage, the rock will not break down, regardless of the duration of the pulse. We employ our knowledge of mechanics to comprehend this issue. For instance, when a small object is placed on a horizontal surface, 10 N of ground friction is exerted onto it. According to the principles of mechanics, a minimum force of 10 N is required to create this effect and cause the object to move. Therefore, for different rocks, the voltage or current generator must be selected appropriately. The electrical parameters of the circuit module also must be selected appropriately, greater than or equal to the minimum breakdown voltage, in order to achieve a better breakdown effect and thereby improve the efficiency of rock fracturing.

### 4.3. Effect of Electrode Spacing on EPB

**Figure 12:**
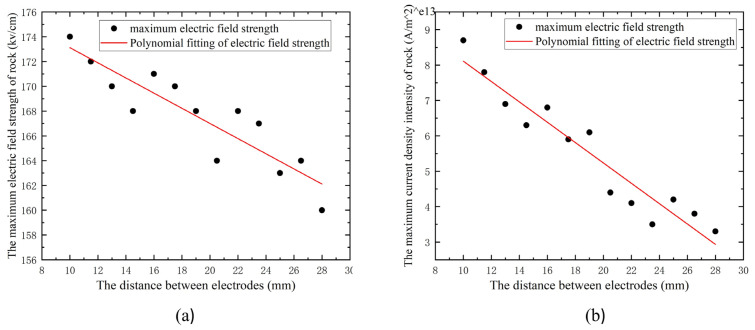
Relationships between electrode spacing and (**a**) maximum electric field and (**b**) maximum current density intensity.

For the purpose of simulating the influence of electrode spacing on EPB, the composition of granite, electrical characteristics, form of electrodes, and position of conductor particles were held constant in the event of total rock disintegration. The intensity and distribution of the electric field were simulated and studied for 13 distances between high and low voltage electrodes. With a step size of −1.5 mm, the high- and low-voltage electrode spacing ranged from 15 mm to 28 mm. As illustrated in Figure 12a, the maximum electric field intensity on the granite surface changes with the electrode spacing. In the agreement with the findings of Changping Li et al. [17], the maximum electric field intensity on the granite surface increases as the electrode spacing decreases, as shown by the graphs. The composition of the granite includes potassium feldspar, plagioclase, quartz, and black mica. Figure 12b depicts the change of current density on the granite surface as a function of electrode spacing, and it can be seen that the maximum current density intensity on the rock surface increases as the electrode spacing decreases.

**Figure 13:**
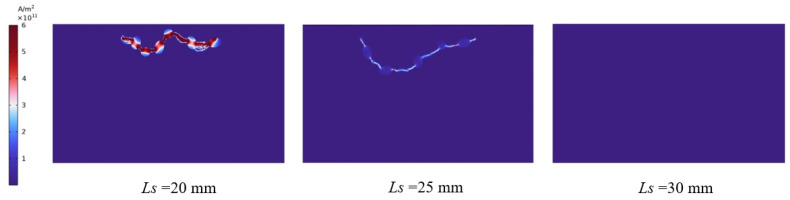
Effect of plasma channel formation at different electrode spacing.

In order to examine the effect of electrode spacing on the creation of plasma channels in greater detail, and in order to observe the electric field and current density distribution in the granite during the EPB process, the results of numerous 5 mm intervals (20 mm, 25 mm, 30 mm) were established in the model. Figure 13 depicts the outcomes of modeling the effect of electrode spacing on the current density distribution within the granite while holding other parameters constant during the EPB process. From these binary relationships, it can be seen that the maximum current density inside the granite grows as the electrode spacing decreases, indicating that the rupture depth of rock is greater in a single discharge. It is evident from the current density distribution that when the spacing is 20 mm, the plasma channel has minimal ability to spread outward and the depth of downward disintegration is only approximately 1/5 of the rock’s diameter. When the electrode spacing is 25 mm, or half the length of the rock, the penetration effect is more in line with actual engineering specifications, achieving a larger penetration effect, the depth approaches one-third of the rock width, and the rock fracturing efficiency is increased. However, when the electrode distance reaches 30 mm, the plasma channel cannot form; hence, extending the electrode distance necessitates a greater electrode voltage for breakdown to be possible. In addition, the electric field intensity within the rock is greatest at the high-voltage electrode, and it decreases with increasing radial distance from the high-voltage electrode. In order to maximize the effectiveness of rock fracturing, the electrode distance should be maintained at half the rock’s length. This leads to the rock fracturing over a broader area, hence increasing the efficiency of rock fracturing.

### 4.4. Effect of Pulse Rise Time on EPB

**Figure 14:**
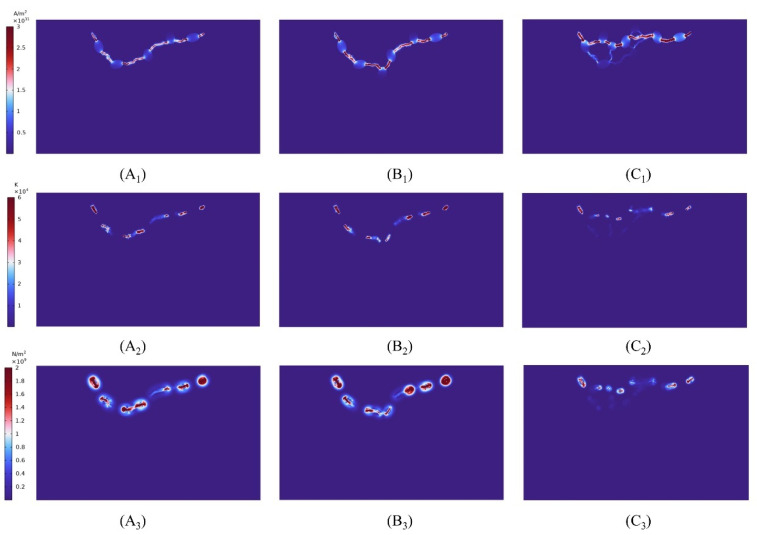
The effect of different pulse rising edge times on EPB, where (**A_1_**,**B_1_**,**C_1_**) represent the distribution of current density intensity, temperature intensity and stress intensity at 200 ns for *t*_11_; where (**A_2_**,**B_2_**,**C_2_**) represent the distribution of current density intensity, temperature intensity and stress intensity at 250 ns for *t*_11_, where (**A_3_**,**B_3_,C_3_**) represent the distribution of current density intensity, temperature intensity and stress intensity at 400 ns for *t*_11_.

First, the peak voltage of the high voltage electrode is maintained at 28 kv, and rock fracture is simulated at pulse durations of 200 ns, 250 ns, and 400 ns, respectively. Figure 14 depicts the law of plasma channel creation within the rock at various pulse rise times and peak voltages. From the temperature distribution, it can be seen that as the pulse time increases, the temperature at the channel decreases slightly compared to that at 200 ns and 400 ns, and the magnitude of the stress is relatively smaller, although both exceed the critical stress magnitude of the rock and both can cause rock fracture. The graphic reveals that the primary plasma channel within the rock at a pulse time of 400 ns differs from that at 200 ns and 400 ns, and that several channels branch off from the main channel. Therefore, in the rock-fracturing project, the pulse period of electrode voltage can be suitably raised to ensure that the rock is entirely shattered.

### 4.5. Heterogeneity of Rock Particles

One of the fundamental aspects of rocks is their heterogeneity, which is mostly expressed in the heterogeneity of the physical and mechanical properties of the different fine structures of rocks, which, in this work, refers to the differences in electrical properties. This heterogeneity of the rock’s microscopic media is the primary cause of the rock’s macroscopic inhomogeneity and nonlinear behavior under external force. Due to the presence of inhomogeneity inside the rock, the production and extension of local microfine cracks result in the formation of macroscopic cracks in the rock. The development process of plasma channels in rocks under the influence of electric pulses has revealed that the channels are always communicated via conductor particles. This may be associated with the electrical conductivity of the particles’ size. We set up conductor particles with varying conductivity diameters for modeling purposes.

**Figure 15:**
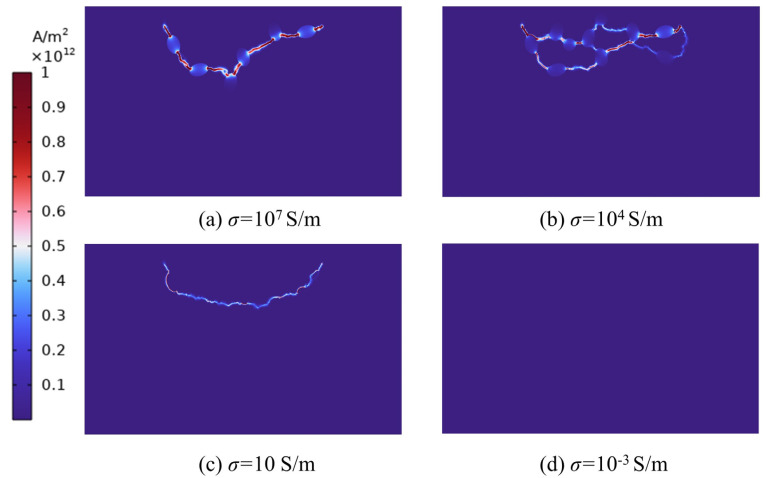
Effect of particles with different electrical conductivity on the formation of plasma channels.

The pulse time is set to 200 ns, the peak pulse of 80 kv, the shape of the electrode and the position of the conductor particles are maintained. Figure 15 displays the simulation results for the conductor particles with varying conductivity. According to the simulation results, the varying conductivity of conductor particles has a significant effect on the creation of plasma channels. Figure 15a,b shows that when the conductivity is high, the plasma channels stretch along the conductor particles, with the possibility of several plasma channels forming. This is the first observation that can be made. When the conductivity of the conductor particles is assumed to be 10, it is evident from Figure 15c that plasma channels do not follow the conductor particles rigidly at this moment. Conductor particles play a significant role in guiding the creation of plasma channels and, to some extent, in determining the rupture course of rocks.

Figure 15d depicts the simulation results when the conductivity is assumed to be 0.001, at which point no plasma channels are created within the rock, and a higher electrode voltage is required to generate ion channels and therefore the rock fractures. When we remove the conductor particles from the model, the simulation results resemble those of Figure 15d, again failing to form plasma channels, at which point it is unlikely that rock fracture will occur. In order to do this, the voltage given to the electrodes was increased from 80 kv to 95 kv, and the results demonstrated that plasma channels also developed within the rock, and that the stresses generated at the channels were sufficient to cause rupture at the channels. This demonstrates that the conductor particles within the rock can not only play a role in guiding the current transmission but can also minimize the electrode voltage necessary for rock breakdown under the same conditions, which provides some benefits for the electric pulse rock breaking.

## 5. Conclusions

Our research examines the electrical response of electric pulse rock breaking based on three modules: the current field, the temperature field, and the mechanical field. We provide a new explanation of the mechanism of the electric pulse rock-fracturing process. More importantly, the model created in our study accurately describes the development process of the plasma channel. This is a practical significance to the research of oil exploration and mineral extraction technology. Our research also allows the prediction of the trend of electric pulse rock fracturing so that the rocks can be properly utilized, especially in mineral extraction engineering, and so that minerals can be fully exploited. In future research and related engineering applications more aspects need to be studied, such as whether some pulse frequencies, pulse widths, etc., also have an impact on the rock-fracturing process. In practical engineering applications, we can use this mechanism to improve the efficiency of electric pulse rock fracturing. In addition, the breakdown characteristics of the material and the dynamic loading of the electric pulse current are examined. The following are the principal conclusions:

(1) The simulation results presented in this paper demonstrate that the temperature distribution and stress distribution are consistent with the location of the current density intensity distribution after the formation of the plasma channel, proving that the plasma channel is the most likely path of subsequent rock fracturing rupture after the completeness of the breakdown;
(2) There is a strong correlation between the distribution of temperature and stress within the rock and the creation of the plasma channel before and after. Prior to the formation of the plasma channel, the highest temperature inside the rock is approximately 10^3^ k and the stress is only 10^−2^ MPa; when the plasma channel is formed, the temperature can reach more than 10^4^ k and the stress can reach 1 GPa, which significantly exceeds the stress intensity of the rock and therefore causes the rock to rupture;
(3) The electrode spacing influences the distribution of the electric field intensity, which in turn controls the likelihood of rock penetration. Keeping all other parameters equal, the shorter the spacing, the easier it is to form plasma channels and break rocks; conversely, increasing the spacing makes it more challenging to break rocks, since the rock’s breakdown field strength may not be attained. When the spacing is maintained at approximately half the length of the rock, modelling findings indicate that the deeper the downward penetration, the greater the effectiveness of rock fracturing;
(4) Finally, our work examines the role that conductor particles play in the electric pulse mechanism. Under the influence of the electrode voltage, the plasma channel will first extend in the direction of the conductor particles due to the high conductivity of the conductor particles compared to that of the rock. In addition, the larger the electrical conductivity of the particles, the more favorable the formation of plasma channels will be.

## Figures and Tables

**Table 1:** The value of the model parameters.

Name	Symbol	Value	Unit
Sample width	Wr	50	mm
Sample height	Hr	25	mm
Electrode gap distance	Ls	25	mm
Electrode voltage	V0	28	kV
Left electrode: Dirichlet BC	-	-	kV
Right electrode: Dirichlet BC	-	-	kV

**Table 2:** Material properties of electrodes and insulating medium.

Material/Property	Conductivity (S/m)	Relative Permittivity	Density (kg/m^3^)	Specific Heat Capacity (J⋅kg^−1^⋅K^−1^)	Thermal Conductivity (W⋅m^−1^⋅K^−1^)
Electrodes	5.7 × 10^7^	1	8960	385	400
Insulating medium	0	4	1150	1700	0.26

**Table 3:** Basic parameters of the rock.

Material Property	Rock	Unit
Conductivity	4	10^−5^ S/m
Relative permittivity	12.0	1
Density	2630	kg/m^3^
Specific heat capacity	711	J⋅kg^−1^⋅K^−1^
Coefficient of thermal expansion	0.75	10^−5^ K^−1^
Thermal conductivity	2.34	W⋅m^−1^⋅K^−1^
Elastic modulus	53.0	GPa
Poisson’s ratio	0.13	1

## Data Availability

Data sharing not applicable.
